# Supplementary material for: Increased random exploration in schizophrenia is associated with inflammation
Source: NPJ Schizophr. 2021 Feb 3;7:6. doi: 10.1038/s41537-020-00133-0 (PMC7859392; doi:10.1038/s41537-020-00133-0)
Supplement: Supplementary file 1 — Supplementary Information [file 41537_2020_133_MOESM1_ESM.pdf]

Supplementary Information

Figure and Table Legends

Supplementary Figure 1. Overview of the bandit task sequence. The three blue squares in every screenshot indicate the individual bandits. After selecting the bandit with the keypad, the points won are displayed in the center of the bandit. RT = Reaction time, ITI: Inter trial interval.

Supplementary Figure 2. Defining directed and random exploration based on the value of the selected bandit (i.e. random exploration equals switching from the best to the worst and directed exploration switching from the best to the second best option) revealed **A)** increased random (t = -2.31, p = 0.019), but not **B)** directed exploration (t = -1.52, p = 0.133) of patients with schizophrenia (SZ) compared to healthy control (HC) participants. * $p < 0.05$. n.s. = non significant.

Supplementary Figure 3. Centrality analysis shows that high sensitivity C-reactive protein (hsCRP) has the highest strength of all immune-parameters included. For a list of abbreviations see **Table S2**.

Supplementary Figure 4. Bootstrap based significance testing of non-zero estimated edge-weights of the network shown in **Fig. 2B**. Non-significant differences between edge weights are indicated by gray boxes, significant differences by black boxes. Significance is based on α = 0.05. The colour of the diagonal boxes (ranging from red to blue) corresponds to the weights of the edges, with red indicating negative and blue positive weights. For a list of abbreviations see **Supplementary Table 2**.

Supplementary Figure 5. Bootstrap based significance testing of node strength for the nodes of the network shown in **Fig. 2B**. Non-significant differences between strength of

two nodes are indicated by gray boxes, significant differences by black boxes. Node strength of each node is presented by the number in the white boxes. For a list of abbreviations see **Supplementary Table 2**.

Supplementary Figure 6. The average correlation between bootstrap centrality measures of the network estimated with all cases and networks in which some cases were dropped. For a list of abbreviations see **Supplementary Table 2**.

Supplementary Figure 7. Bootstrapped 95% confidence intervals for estimated edge weights of the network shown in **Fig. 2B**. For a list of abbreviations see **Supplementary Table 2**.

Supplementary Table 1. Exploitation and random exploration correlate with the Positive and Negative Syndrome Scale (PANSS) disorganized factor but not with PANSS negative, positive or depressive factor or cognitive score. There are no associations between task parameters and chlorpromazine equivalents.

Supplementary Table 2. Abbreviations of immune parameters.

Supplementary Table 3. Group differences of measured peripheral immune parameters. Units: hsCRP: mg/l; immune parameters: normalized protein expression (NPX). For a list of abbreviations see **Supplementary Table 2**. Statistics: [1]Student's t-test. [2]Mann-Whitney U test.

Supplementary Table 4. Significant correlation between random exploration with high sensitivity C-reactive protein (hsCRP), but not with TRAIL or CCL20.

Supplementary Figures

Supplementary Figure 1

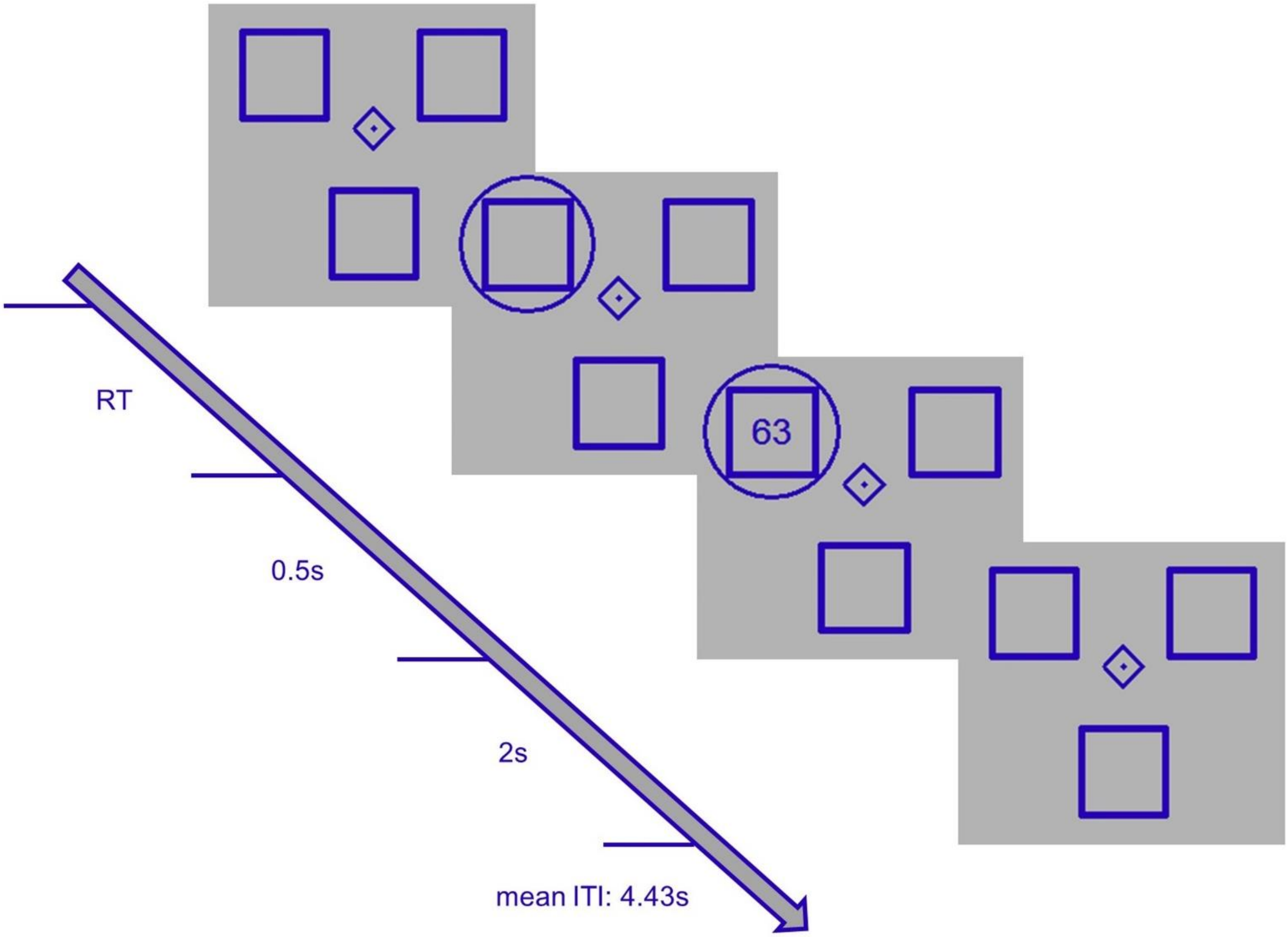

Supplementary Figure 2

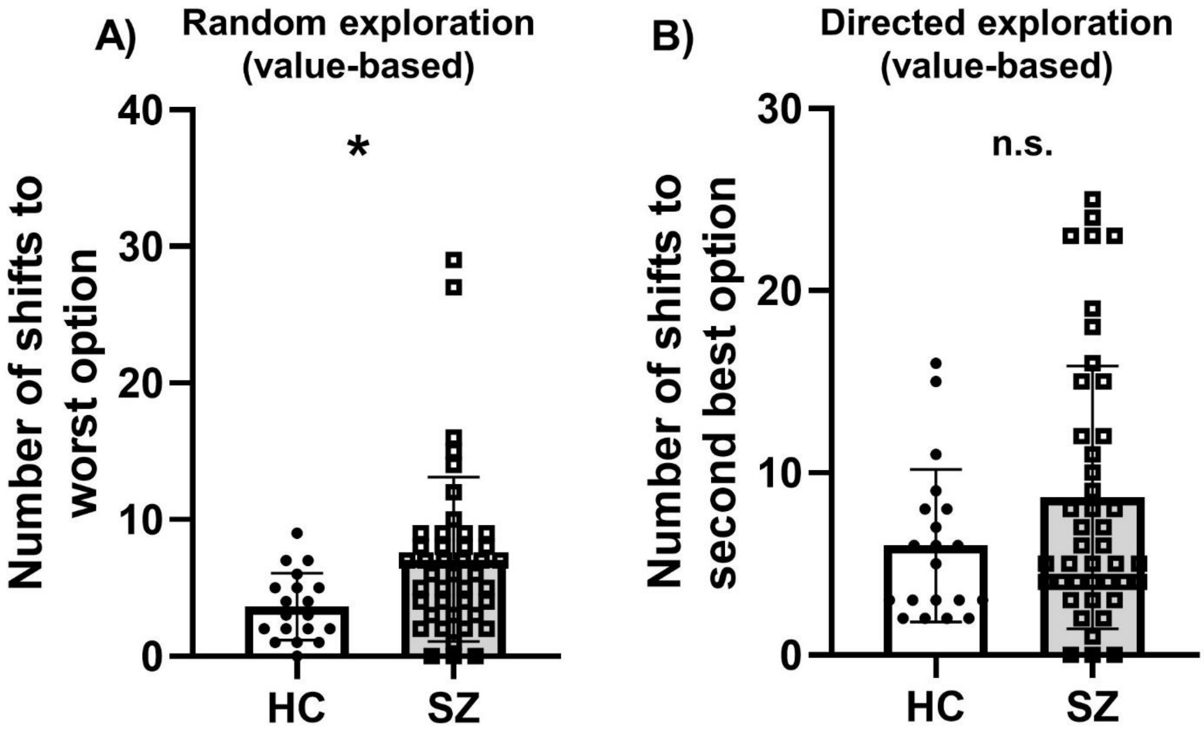

Supplementary Figure 3

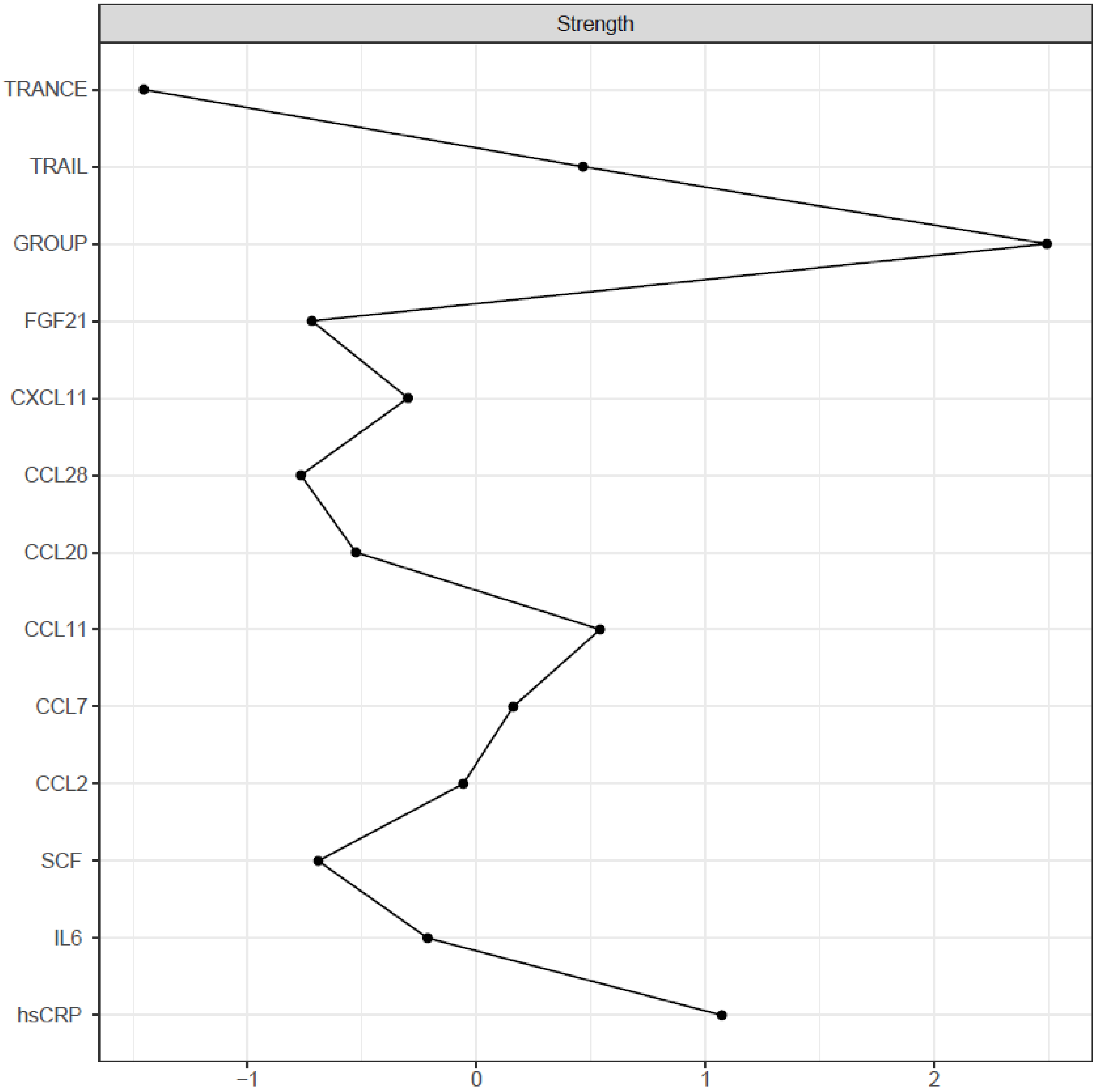

Supplementary Figure 4

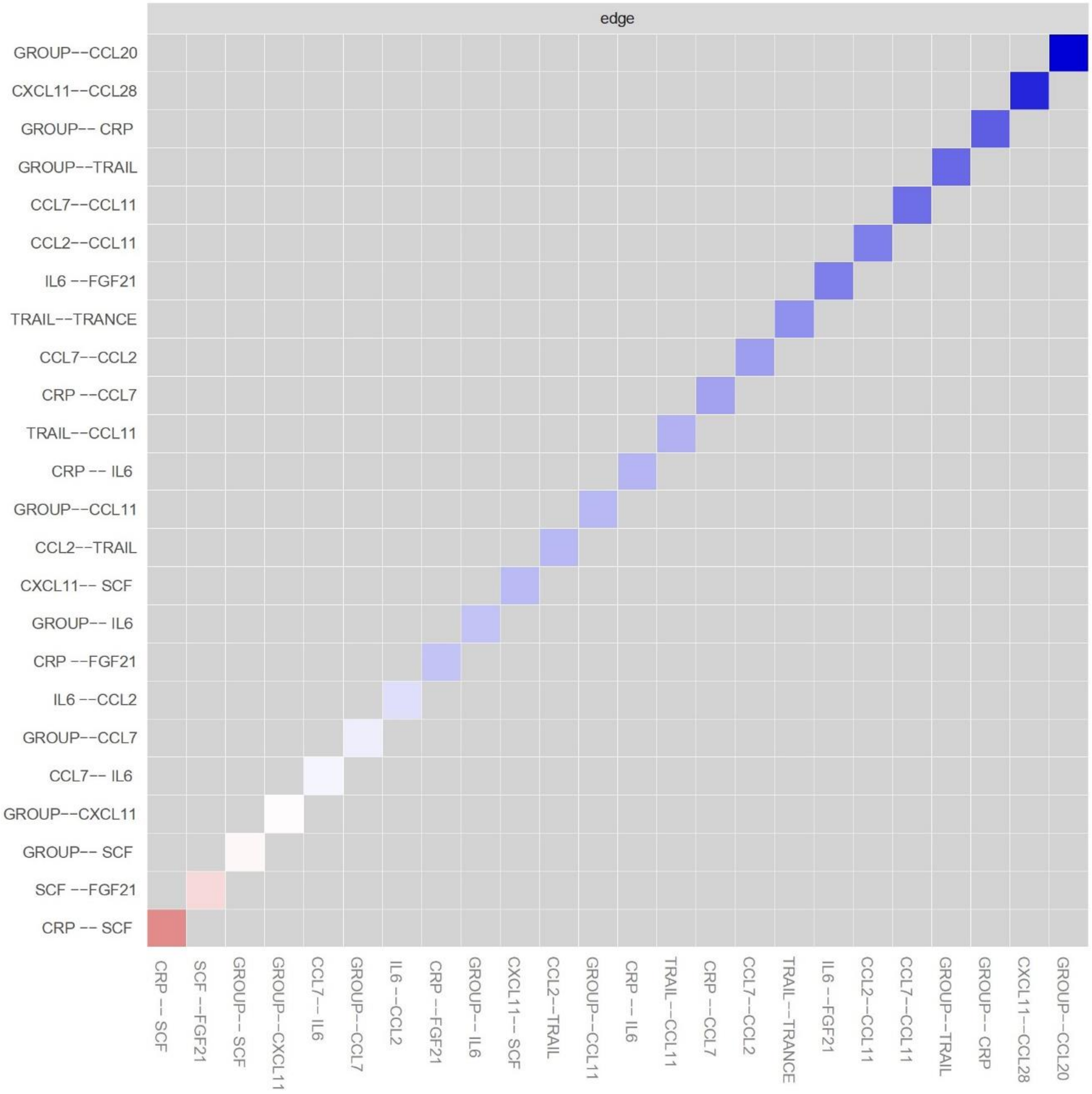

Supplementary Figure 5

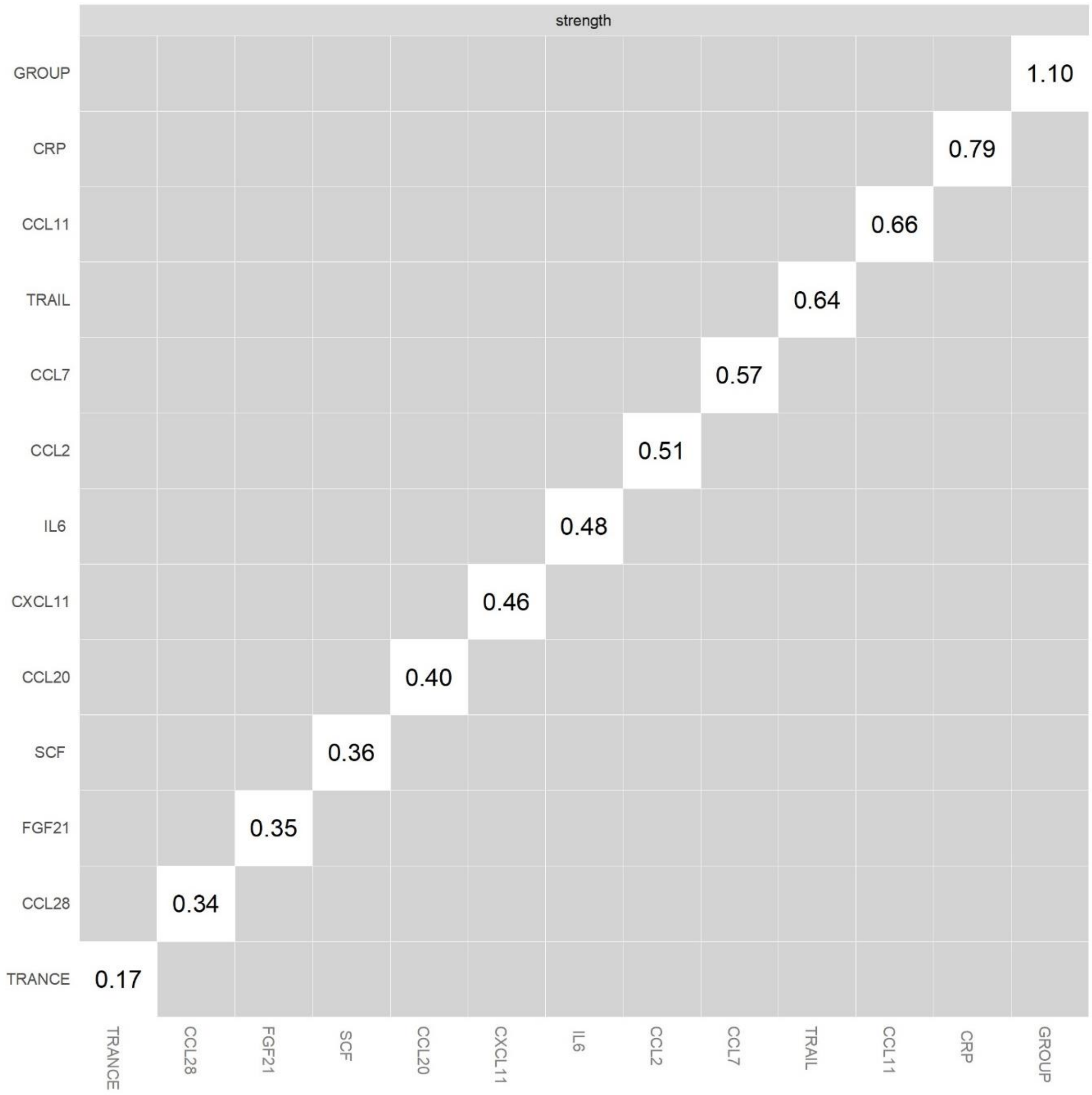

Supplementary Figure 6

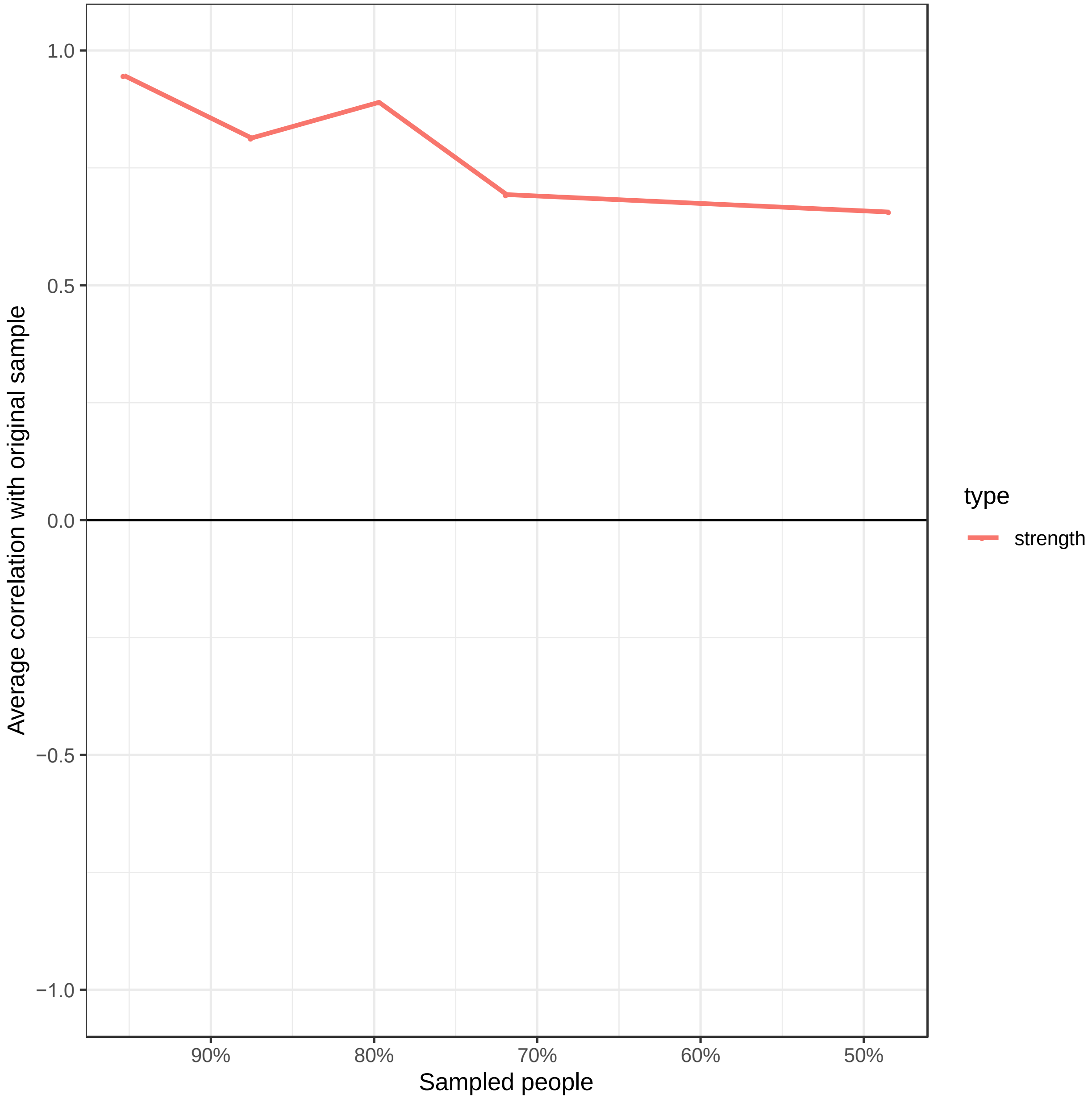

Supplementary Figure 7

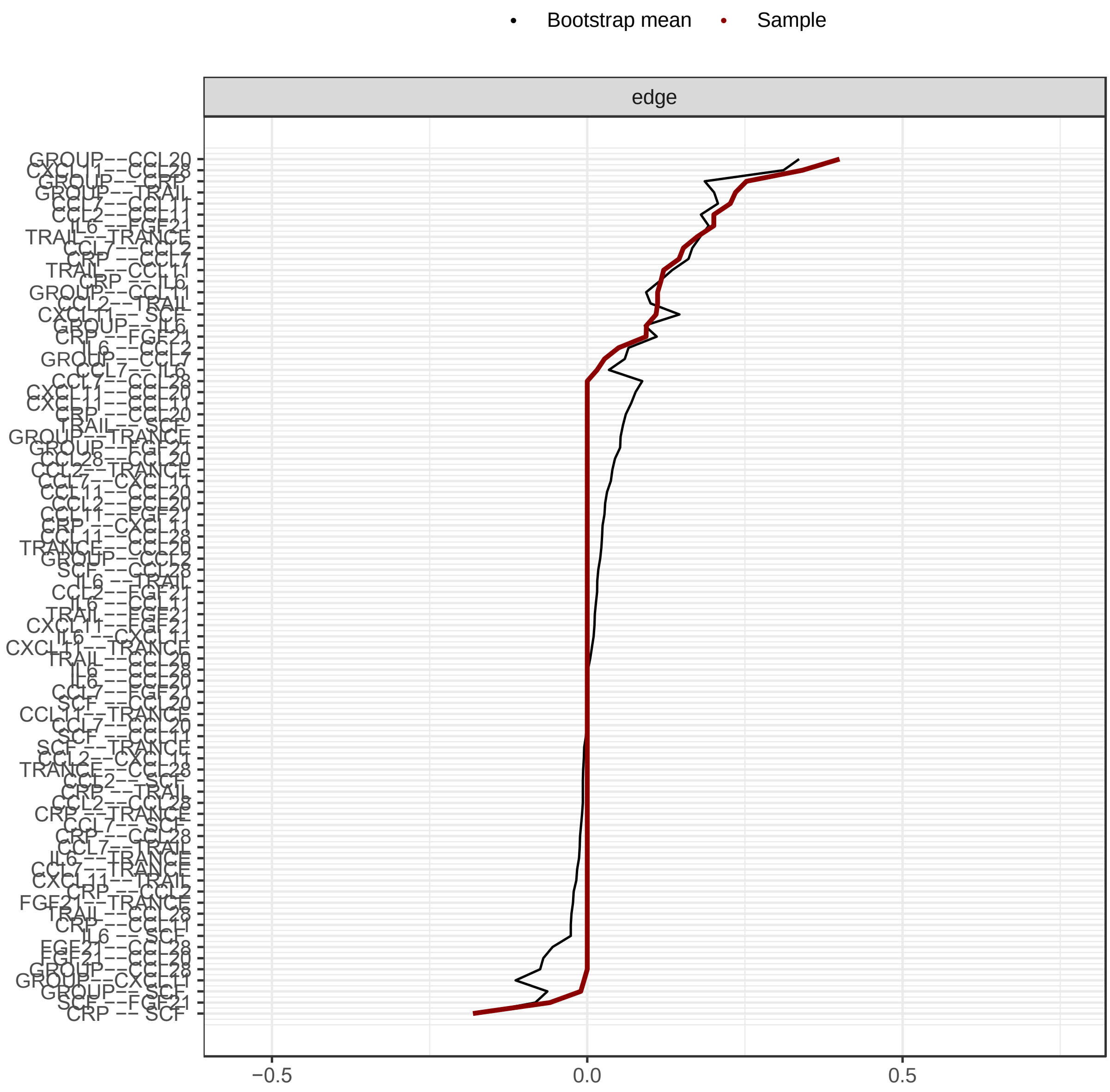

Supplementary Tables

Correlations between task-parameters and psychopathology, composite cognitive score and chlorpromazine equivalents in patients with SZ

**Supplementary Table 1**	PANSS negative factor	PANSS positive factor	PANSS disorganized factor	PANSS depressive factor	Composite cognitive score	Chlorpro-mazine equivalents
Total Points earned	r(s) = - 0.07, *p* = 0.678	r(s) = -0.09, *p* = 0.582	r(s) = -0.30, *p* = 0.055	r(s) = 0.23, *p* = 0.141	r(s) = 0.07, *p* = 0.654	r(s) = -0.21, *p* = 0.172
Exploitation	r(s) = - 0.09, *p* = 0.558	r(s) = 0.15, *p* = 0.328	**r(s) = -0.36, *p* = 0.018**	r(s) = 0.105, *p* = 0.501	r(s) = 0.16, *p* = 0.303	r(s) = -0.22, *p* = 0.159
Random exploration	r(s) = 0.14, *p* = 0.375	r(s) = -0.17, *p* = 0.291	**r(s) = 0.31, *p* = 0.045**	r(s) = -0.07, *p* = 0.644	r(s) = -0.18, *p* = 0.454	r(s) = 0.23, *p* = 0.132
Directed exploration	r(s) = -0.01, *p* = 0.967	r(s) = -0.28, *p* = 0.074	r(s) = 0.27, *p* = 0.078	r(s) = -0.15, *p* = 0.337	r(s) = 0.01, *p* = 0.935	r(s) = 0.15, *p* = 0.338

List of abbreviations of inflammatory parameters

**Supplementary Table 2**	
**Abbreviation**	**Legend**
ADA	Adenosine Deaminase
AXIN1	Axin-1
Beta-NGF	Beta-nerve growth factor
CASP-8	Caspase-8
CCL	C-C motif chemokine
CD	Cluster of differentiation
CDCP1	CUB domain-containing protein 1
hsCRP	High sensitive C-reactive protein
CSF-1	Colony-stimulating factor 1
CST5	Cystatin-D
CXCL	C-X-C motif chemokine
DNER	Delta and notch-like epidermal growth factor-related receptor
EN-RAGE	Protein S100-A12
Flt3L	Fms-related tyrosine kinase 3 ligand
FGF	Fibroblast growth factor
GDNF	Glial cell line-derived neurotrophic factor
HGF	Hepatocyte growth factor
IL	Interleukin
IL-R	Interleukin receptor
LAPTGF	Latency-associated peptide transforming growth factor beta-1
Lif-R	Leukemia inhibitory factor receptor
MMP	Matrix metalloproteinase
NT-3	Neurotrophin-3
OPG	Osteoprotegerin
OSM	Oncostatin-M
PD-L1	Programmed cell death 1 ligand 1
SCF	Stem cell factor
SLAMF1	Signaling lymphocytic activation molecule family member 1
SIRT2	SIR2-like protein 2
ST1A1	Sulfotransferase 1A1
STAMPB	STAM-binding protein
TGF	Transforming growth factor
TNFB	Tumor necrosis factor beta
TNFRSF9	Tumor necrosis factor receptor superfamily member 9
TNFSF14	Tumor necrosis factor ligand superfamily member 14
TRANCE	Tumor necrosis factor-related activation-induced cytokine
TWEAK	Tumor necrosis factor (ligand) superfamily, member 12
TRAIL	Tumor necrosis factor related apoptosis-inducing ligand
uPA	Urokinase-type plasminogen activator
VEGFA	Vascular endothelial growth factor

**Supplementary Table 3**	**HC** (n = 19)	**SZ** (n = 45)	**Test statistics**	
			**(t^1/U^2)**	***p***
ADA	3.96 (0.61)	3.75 (0.62)	U = 272.5	*p* = 0.157
AXIN1	5.13 (2.07)	5.09 (1.40)	U = 348.0	*p* = 0.880
Beta-NGF	1.54 (0.24)	1.44 (0.17)	t = 1.899	*p* = 0.063
CASP-8	1.95 (1.26)	1.55 (0.87)	U = 324.5	*p* = 0.586
CCL2	9.54 (0.37)	9.78 (0.43)	t = -2.046	***p* = 0.045**
CCL3	4.20 (0.50)	4.35 (0.56)	U = 288.0	*p* = 0.248
CCL4	6.12 (0.48)	6.18 (0.65)	t = -0.317	*p* = 0.753
CCL7	0.82 (0.37)	1.15 (0.53)	t = -2.234	***p* = 0.030**
CCL8	7.89 (0.59)	7.62 (0.74)	U = 265.5	*p* = 0.126
CCL11	7.16 (0.38)	7.52 (0.48)	t = -2.741	***p* = 0.008**
CCL13	3.49 (0.74)	3.40 (0.76)	U = 327.0	*p* = 0.616
CCL19	8.63 (1.09)	8.58 (0.62)	U = 334.5	*p* = 0.706
CCL20	4.55 (0.57	5.55 (1.26)	U = 158.5	***p* < 0.001**
CCL23	9.63 (0.39)	9.49 (0.48)	t = 1.085	*p* = 0.282
CCL25	5.74 (0.65)	5.96 (0.55)	t = -1.320	*p* = 0.192
CCL28	1.10 (0.43)	0.89 (0.47)	U = 183.5	***p* = 0.003**
CD5	4.77 (0.27)	4.75 (0.42)	U = 326.0	*p* = 0.604
CD6	4.45 (0.45)	4.19 (0.50)	t = 1.837	*p* = 0.071
CD40	10.14 (0.68)	9.91 (0.62)	U = 281.5	*p* = 0.206
CD244	6.06 (0.63)	5.84 (0.56)	U = 271.0	*p* = 0.150
CDCP1	2.21 (0.51)	2.45 (0.53)	U = 259.5	*p* = 0.103
hsCRP	0.89 (0.63)	2.18 (1.80)	U = 243.5	***p* = 0.007**
CSF-1	7.78 (0.25)	7.76 (0.23)	t = 0.232	*p* = 0.818
CST5	5.82 (0.49)	5.70 (0.57)	U = 297.5	*p* = 0.319
CXCL1	8.77 (1.02)	8.59 (0.92)	t = 0.648	*p* = 0.520
CXCL5	11.28 (1.47)	10.96 (1.16)	U = 335.0	*p* = 0.713
CXCL6	8.19 (1.27)	7.85 (1.06)	U = 309.5	*p* = 0.427
CXCL9	6.82 (1.05)	6.63 (0.54)	U = 350.0	*p* = 0.907
CXCL10	7.75 (1.04)	7.42 (0.75)	U = 287.5	*p* = 0.245
CXCL11	7.85 (1.03)	7.23 (1.15)	U = 223.0	***p* = 0.026**
CX3CL1	4.92 (0.35)	4.81 (0.35)	t = 1.078	*p* = 0.286
DNER	7.94 (0.24)	7.99 (0.29)	t = -0.532	*p* = 0.597
EN-RAGE	1.64 (0.87)	1.76 (0.75)	U = 325.5	*p* = 0.598
Flt3L	8.48 (0.52)	8.54 (0.54)	t = -0.405	*p* = 0.687
FGF-5	1.00 (0.20)	0.93 (0.20)	t = 1.225	*p* = 0.226
FGF-19	7.95 (0.93)	7.67 (0.83)	t = 1.133	*p* = 0.262
FGF-21	4.56 (1.35)	5.34 (1.20)	t = - 2.186	***p* = 0.033**
FGF-23	1.85 (0.51)	1.95 (0.36)	U = 279.5	*p* = 0.195
GDNF	1.38 (0.41)	1.33 (0.43)	U = 303.5	*p* = 0.371
HGF	7.70 (0.34)	7.82 (0.36)	t = -1.203	*p* = 0.234
IL-6	2.47 (0.51)	3.01 (0.91)	t = -2.308	***p* = 0.025**
IL-7	3.91 (0.93)	3.62 (0.87)	U = 295.0	*p* = 0.299
IL-8	5.18 (0.56)	5.27 (0.57)	t = -0.596	*p* = 0.554
IL-10	2.73 (1.28)	2.67 (0.52)	U = 281.5	*p* = 0.206
IL-10RA	1.44 (0.98)	1.36 (1.29)	U = 285.0	*p* = 0.228
IL-10RB	6.38 (0.25)	6.43 (0.26)	t = -0.673	*p* = 0.504
IL-12B	4.10 (0.66)	4.00 (0.78)	t = 0.448	*p* = 0.656
IL-15RA	0.11 (0.18)	0.11 (0.20)	U = 350.0	*p* = 0.907
IL-17C	1.38 (0.42)	1.61 (0.56)	t = -1.547	*p* = 0.127
IL-18	7.49 (0.47)	7.54 (0.47)	t = -0.369	*p* = 0.713
IL-18R1	6.66 (0.46)	6.71 (0.33)	t = -0.465	*p* = 0.643
K4E-BP1	8.69 (1.50)	8.55 (1.08)	t = 0.409	*p* = 0.684
LAPTGF	7.57 (0.72)	7.23 (0.60)	U = 250.0	*p* = 0.073
Lif-R	2.30 (0.25)	2.29 (0.23)	U = 347.5	*p* = 0.874
MMP-1	11.98 (1.46)	11.68 (1.17)	t = 0.823	*p* = 0.414
MMP-10	5.34 (0.46)	5.46 (0.68)	U = 344.0	*p* = 0.828
NT-3	1.82 (0.34)	1.81 (0.39)	U = 331.0	*p* = 0.663
OPG	10.31 (0.38)	10.15 (0.29)	t = 1.743	*p* = 0.087
OSM	2.31 (0.70)	2.47 (0.98)	t = -0.576	*p* = 0.567
PD-L1	3.58 (0.36)	3.52 (0.34)	t = 0.576	*p* = 0.567
SCF	9.65 (0.39)	9.40 (0.42)	t = 2.125	***p* = 0.038**
SLAMF1	1.27 (0.46)	1.15 (0.37)	t = 1.067	*p* = 0.291

SIRT2	4.37 (2.11)	4.11 (1.47)	U = 352.0	*p* = 0.933
ST1A1	2.96 (1.35)	2.68 (1.00)	U = 316.0	*p* = 0.493
STAMPB	5.59 (1.80)	5.35 (1.25)	U = 352.0	*p* = 0.933
TGF-alpha	2.34 (0.25)	2.38 (0.26)	t = -0.458	*p* = 0.649
TNF-beta	3.57 (0.31)	3.58 (0.45)	t = -0.091	*p* = 0.928
TNFRSF9	5.72 (0.40)	5.87 (0.40)	t = -1.282	*p* = 0.205
TNFSF14	4.53 (0.91)	4.43 (0.70)	U = 349.0	*p* = 0.893
TRAIL	7.94 (0.44)	8.31 (0.24)	t = -4.164	***p* < 0.001**
TRANCE	4.06 (0.74)	4.47 (0.67)	t = -2.087	***p* = 0.041**
TWEAK	9.33 (0.42)	9.20 (0.43)	t = 1.076	*p* = 0.286
uPA	10.24 (0.24)	10.29 (0.30)	t = -0.548	*p* = 0.586
VEGFA	9.31 (0.44)	9.33 (0.50)	U = 335.0	*p* = 0.719

Correlations between hsCRP, TRAIL and CCL20 with bandit task parameters in patients with SZ

**Supplementary Table 4**	Total points earned (Reward)	Continuously best (N) (Exploitation)	Shifts to less informative option (N) (Random Exploration)	Shifts to more informative option (N) (Directed Exploration)
hsCRP	r(s) = -0.19, *p* = 0.225	r(s) = -0.27, *p* = 0.081	**r(s) = 0.40, *p* = 0.008**	r(s) = 0.17, *p* = 0.284
TRAIL	r(p) = -0.11, *p* = 0.517	r(p) = -0.12, *p* = 0.445	r(s) = 0.19, *p* = 0.248	r(s) = 0.16, *p* = 0.326
CCL20	r(s) = -0.21, *p* = 0.196	r(s) = 0.06, *p* = 0.737	r(s) = 0.10, *p* = 0.557	r(s) = 0.13, *p* = 0.414
