# Supplementary material for: Increased random exploration in schizophrenia is associated with inflammation
Source: NPJ Schizophr. 2021 Feb 3;7:6. doi: 10.1038/s41537-020-00133-0 (PMC7859392; doi:10.1038/s41537-020-00133-0)
Supplement: Supplementary file 2 — Reporting summary [file 41537_2020_133_MOESM2_ESM.pdf]

nature research

Corresponding author(s): NPJSCHZ-00503R1

Last updated by author(s): 18 11 2020

Reporting Summary

Nature Research wishes to improve the reproducibility of the work that we publish. This form provides structure for consistency and transparency in reporting. For further information on Nature Research policies, see our Editorial Policies and the Editorial Policy Checklist.

Statistics

For all statistical analyses, confirm that the following items are present in the figure legend, table legend, main text, or Methods section.

n/a	Confirmed	
☐	☒	The exact sample size (*n*) for each experimental group/condition, given as a discrete number and unit of measurement
☐	☒	A statement on whether measurements were taken from distinct samples or whether the same sample was measured repeatedly
☐	☒	The statistical test(s) used AND whether they are one- or two-sided<br>*Only common tests should be described solely by name; describe more complex techniques in the Methods section.*
☐	☒	A description of all covariates tested
☐	☒	A description of any assumptions or corrections, such as tests of normality and adjustment for multiple comparisons
☐	☒	A full description of the statistical parameters including central tendency (e.g. means) or other basic estimates (e.g. regression coefficient) AND variation (e.g. standard deviation) or associated estimates of uncertainty (e.g. confidence intervals)
☐	☒	For null hypothesis testing, the test statistic (e.g. F, t, r) with confidence intervals, effect sizes, degrees of freedom and P value noted<br>*Give P values as exact values whenever suitable.*
☒	☐	For Bayesian analysis, information on the choice of priors and Markov chain Monte Carlo settings
☒	☐	For hierarchical and complex designs, identification of the appropriate level for tests and full reporting of outcomes
☐	☒	Estimates of effect sizes (e.g. Cohen's *d*, Pearson's *r*), indicating how they were calculated

Our web collection on statistics for biologists contains articles on many of the points above.

Software and code

Policy information about availability of computer code

Data collection	Interviews were recorded as audiofiles and were transcribed.
Data analysis	Open-source PRAAT software was used for acoustic analyses. Code for speech preprocessing (WordNet lemmatizer) and POS-Tag (Pen Tree Bank) is available open access through the NLTK (http://www.nltk.org/).

For manuscripts utilizing custom algorithms or software that are central to the research but not yet described in published literature, software must be made available to editors and reviewers. We strongly encourage code deposition in a community repository (e.g. GitHub). See the Nature Research guidelines for submitting code & software for further information.

Data

Policy information about availability of data

All manuscripts must include a data availability statement. This statement should provide the following information, where applicable:
- Accession codes, unique identifiers, or web links for publicly available datasets
- A list of figures that have associated raw data
- A description of any restrictions on data availability

The data that support the findings of this study are available from the corresponding author upon request.

Field-specific reporting

Please select the one below that is the best fit for your research. If you are not sure, read the appropriate sections before making your selection.

☐ Life sciences ☒ Behavioural & social sciences ☐ Ecological, evolutionary & environmental sciences

For a reference copy of the document with all sections, see nature.com/documents/nr-reporting-summary-flat.pdf

Behavioural & social sciences study design

All studies must disclose on these points even when the disclosure is negative.

Study description	Quantitative cross-section
Research sample	Participants met criteria for the "Clinical High Risk" (CHR) syndrome of attenuated positive symptoms, as assessed with the Structured Interview for Psychosis-Risk Syndromes/Scale of Psychosis-Risk Symptoms. Participants were 33 CHR individuals with mean(SD) age = 21(4) years, who were 1/3 female and ethnically diverse (36% Caucasian). 24% of participants were prescribed medications (9% antipsychotics, 21% antidepressants)
Sampling strategy	Subsequent participants in a clinical research program who provided consent for participation. No prior sample size was estimated. The sample is equivalent to that described in a prior NPJ Schizophrenia manuscript entitled "Automated analysis of free speech predicts psychosis onset in high-risk youths": https://www.nature.com/articles/npjschz201530
Data collection	Data were collected via audiorecording. Only interviewers and research participants were present. Interviewers were blind to these study hypotheses. These methods of data collection are identical to that described in a prior NPJ Schizophrenia manuscript entitled "Automated analysis of free speech predicts psychosis onset in high-risk youths": https://www.nature.com/articles/npjschz201530
Timing	2007 to 2011
Data exclusions	No data were excluded from analysis.
Non-participation	This was a cross-sectional study so dropout was minimal.
Randomization	No randomization occurred.

Reporting for specific materials, systems and methods

We require information from authors about some types of materials, experimental systems and methods used in many studies. Here, indicate whether each material, system or method listed is relevant to your study. If you are not sure if a list item applies to your research, read the appropriate section before selecting a response.

Materials & experimental systems

n/a	Involved in the study	
☒	☐	Antibodies
☒	☐	Eukaryotic cell lines
☒	☐	Palaeontology and archaeology
☒	☐	Animals and other organisms
☐	☒	Human research participants
☐	☒	Clinical data
☒	☐	Dual use research of concern

Methods

n/a	Involved in the study	
☒	☐	ChIP-seq
☒	☐	Flow cytometry
☒	☐	MRI-based neuroimaging

Human research participants

Policy information about studies involving human research participants

Population characteristics	Participants met criteria for the "Clinical High Risk" (CHR) syndrome of attenuated positive symptoms, as assessed with the Structured Interview for Psychosis-Risk Syndromes/Scale of Psychosis-Risk Symptoms. Participants were 33 CHR individuals with mean(SD) age = 21(4) years, who were 1/3 female and ethnically diverse (36% Caucasian). 24% of participants were prescribed medications (9% antipsychotics, 21% antidepressants)
Recruitment	Participants were help-seeking individuals in a psychosis risk clinical research program.
Ethics oversight	New York State Psychiatric Institute IRB; Icahn School of Medicine at Mount Sinai IRB

Note that full information on the approval of the study protocol must also be provided in the manuscript.

Clinical data

Policy information about clinical studies

All manuscripts should comply with the ICMJE guidelines for publication of clinical research and a completed CONSORT checklist must be included with all submissions.

Clinical trial registration	Not applicable.
Study protocol	Please see prior NPJ Schizophrenia manuscript entitled "Automated analysis of free speech predicts psychosis onset in high-risk youths": https://www.nature.com/articles/npjschz201530
Data collection	Speech was elicited through open-ended narrative interviews12, in which participants were instructed to discuss their lives broadly; interviews were conducted using qualitative methods13 meant to maximize the amount of narrative speech by the person interviewed with interviewers interjecting only to encourage the participant to speak further. Interviewers were trained in qualitative interviewing by an expert in phenomenological research methods8. Over approximately one hour, participants were encouraged to describe their experience, its impact on them, and their expectations for the future. Negative and other symptoms were assessed by PhD raters using the Structured Interview for Psychosis-Risk Syndromes/Scale of Psychosis-Risk Syndromes (SIPS/SOPS)11, separate from narrative interviews.
Outcomes	Negative symptoms were assessed using the Structured Interview for Psychosis Risk Syndromes. Linguistic variables were defined using part-of-speech tagging. Pause behavior variables were extracted using PRAAT.
